# Dynamics of Action Potential Initiation in the GABAergic Thalamic Reticular Nucleus In Vivo

**DOI:** 10.1371/journal.pone.0030154

**Published:** 2012-01-18

**Authors:** Fabián Muñoz, Pablo Fuentealba

**Affiliations:** 1 Departamento de Psiquiatría, Centro de Investigaciones Médicas y Centro Interdisciplinario de Neurociencia, Escuela de Medicina, Pontificia Universidad Católica de Chile, Santiago, Chile; 2 Fundació Sant Joan de Déu, Edifici Docent, Esplugues del Llobregat, Barcelona, Spain; Consejo Superior de Investigaciones Cientificas - Instituto Cajal, Spain

## Abstract

Understanding the neural mechanisms of action potential generation is critical to establish the way neural circuits generate and coordinate activity. Accordingly, we investigated the dynamics of action potential initiation in the GABAergic thalamic reticular nucleus (TRN) using in vivo intracellular recordings in cats in order to preserve anatomically-intact axo-dendritic distributions and naturally-occurring spatiotemporal patterns of synaptic activity in this structure that regulates the thalamic relay to neocortex. We found a wide operational range of voltage thresholds for action potentials, mostly due to intrinsic voltage-gated conductances and not synaptic activity driven by network oscillations. Varying levels of synchronous synaptic inputs produced fast rates of membrane potential depolarization preceding the action potential onset that were associated with lower thresholds and increased excitability, consistent with TRN neurons performing as coincidence detectors. On the other hand the presence of action potentials preceding any given spike was associated with more depolarized thresholds. The phase-plane trajectory of the action potential showed somato-dendritic propagation, but no obvious axon initial segment component, prominent in other neuronal classes and allegedly responsible for the high onset speed. Overall, our results suggest that TRN neurons could flexibly integrate synaptic inputs to discharge action potentials over wide voltage ranges, and perform as coincidence detectors and temporal integrators, supported by a dynamic action potential threshold.

## Introduction

Brain activity is structured by the dynamic encoding and processing of information in neuronal circuits. The cellular components of neural circuits continuously transform varying spatiotemporal patterns of synaptic inputs into trains of action potentials that support network operations and communication [1]. Therefore, the precise timing and pattern of action potentials is critical for the appropriate flow and processing of information in the neural network. A key component in the regulation of information flow is the mechanism of generation of action potentials, which is precisely determined by biophysical mechanisms [2]. It follows that the voltage threshold of action potentials and its variability is highly relevant for it directly affects the spike-timing and temporal precision of synaptic transmission, and minor modifications of the action potential-generating mechanism can qualitatively change the nature of neuronal encoding.

An important component of the temporal sensitivity of cortical neurons derives from the voltage-gated conductances that underlie the action potential [2]. Thus, in some neuronal classes the voltage threshold for spike generation depends on the rate of preceding membrane depolarization [3], [4], [5], [6]. This effect is determined by the gating kinetics of sodium and potassium channels [2], [7], [8], which enhance the sensitivity to rapid depolarizations. Consequently, in some neuronal populations the voltage threshold for spiking is lower and the sensitivity higher when the membrane potential depolarizes rapidly [3], [4]. Accordingly, the spike-generating mechanism has been proposed to amplify coincident inputs, because rapid depolarizations may arise in response to synchronous synaptic inputs. This suggests that some neuronal populations can perform as temporal integrators [3], [9].

Not much information is available about action potential initiation in the thalamus, where neuronal populations exhibit distinct activity patterns, anatomical structure and connectivity [10]. Here, we have studied the in vivo dynamics of action potential initiation in the GABAergic thalamic reticular nucleus (TRN) of adult cats, in order to preserve natural spike timing, patterns of synaptic activity, and intact anatomical structure. The TRN is a key structure in the thalamocortical system as it intersects and regulates synaptic communication between the thalamus and the neocortex, receiving axon collaterals from both structures [11]. The TRN is concerned with most -if not all- functional modalities [12], it has been critically implicated in regulating attention [13], [14], sleep cycle [15], [16], electrical rhythms [11], synaptic plasticity [17], [18] and setting global activity patterns and precise spike-timing through tight GABAergic control of the relay thalamocortical nuclei in health and disease [11].

We report a wide operational spike threshold range, rapid onset and steep initiation of action potentials, and the unexpected voltage-dependent onset speed of action potentials in TRN neurons in vivo, a phenomenon that seemingly depends on the effective density of sodium channels. We previously found that TRN neurons can operate as temporal integrators, due to a long voltage-dependent membrane time constant in the cortically evoked synaptic responses [19]. Here, we also describe a dynamic, wide range of voltage thresholds for spike generation as a function of the rate of membrane depolarization, which suggests that TRN neurons can also perform as coincidence detectors, a function that is considered to be incompatible with effective temporal integration [20], [21]. Thus, our results present a general description of the dynamics of action potential initiation in the thalamic reticular nucleus in vivo, which will contribute to the future understanding of the encoding and processing of information in thalamocortical circuits.

## Results

**Figure 1:**
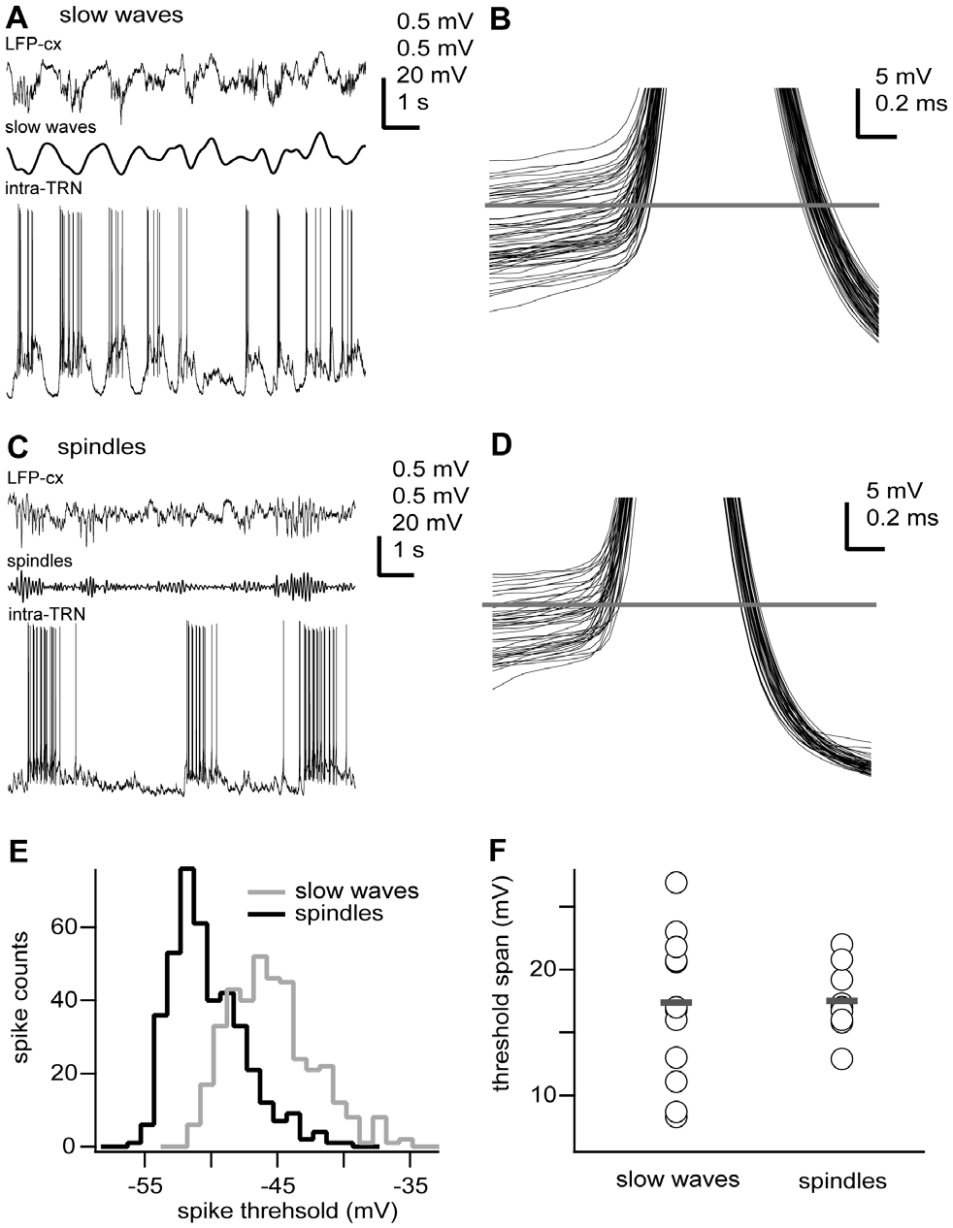
Action potentials of TRN neurons exhibit similarly large threshold voltage range during different network oscillations. **A, C,** In vivo intracellular recordings (intra) from two TRN neurons during either slow oscillations (ketamine-xylazine anaesthesia), A) or spindle oscillations (pentobarbital anaesthesia), C) recorded in the neocortex. **B, D,** Superposition of all action potentials (clipped) depicted on the left column (A, n = 61; C, n = 44) showing the variability in voltage threshold. Horizontal line shows mean voltage threshold for the whole recording epoch (B, −47.7 mV; D, −52.3 mV). Spikes were arbitrarily clipped for illustration purposes only. **E,** Distribution of spike thresholds for neurons shown in A and C (slow oscillations, n = 381 spikes; spindle oscillations, n = 633 spikes). **F,** Action potential voltage threshold range for all recorded neurons (slow waves, ketamine-xylazine anaesthesia; spindles, pentobarbital anaesthesia). Circles and grey bars show individual values and means, respectively. Means were not significantly different (p = 0.9089). Intracellular recording (intra-TRN, 0.1 Hz–20 kHz), cortical local field potential (LFP-cx, 0.1 Hz–20 kHz), slow waves (filtered LFP, 0.1–2 Hz), spindles (filtered LFP, 7–15 Hz). Scale bars: A,C, LFP-cx 0.5 mV, slow waves 0.5 mV, spindles 0.5 mV, intra-TRN 20 mV, horizontal 1 s; B,D, vertical 5 mV, horizontal 0.2 ms. Some portions of raw data (intracellular voltage recordings) from the cells in this figure have been used in previous publications (see Methods).

TRN neurons (n = 27) were intracellularly recorded and electrophysiologically identified by their characteristic low-threshold spikes, high-frequency burst discharge and accelerando-decelerando firing pattern [22]. Stable membrane potential recordings showed that the somatic voltage threshold for action potential initiation was not constant across individual action potentials during the same recording epoch (Fig. 1B,C). In fact, the action potential voltage thresholds spanned a large range (17.48±4.75 mV, n = 22). Such variability could be similarly detected during distinctive network oscillations induced by different anaesthetics (Fig. 1A,C). Indeed, we distinguished two distinct network oscillations in our anesthetised preparations: a slow-wave synchronized state, characterized by large-amplitude, very low-frequency (<1 Hz) LFP patterns, present prominently in animals anaesthetised with ketamine-xylazine [23]; and a spindle-oscillation synchronized state, characterized by large-amplitude, low-frequency (10–15 Hz) LFP patterns, detected in animals anaesthetised with pentobartital [24].

The standard deviation of each voltage threshold distribution provides a measure of spike threshold variation [3]. Thus, the variability in action potential voltage threshold of neurons during slow oscillations (ketamine-xylazine anaesthesia, 2.96±0.87 mV, n = 13) was not different to the one detected during spindle oscillations (pentobarbital anaesthesia, 2.72±0.34 mV, n = 9; p = 0.6585), even though the network rhythms were distinctively different (Fig. 1B,D). Similarly, the voltage threshold values spanned a similar range for both oscillatory regimes (mean values from threshold range distributions were not statistically different: 17.45±5.86 mV, n = 13, and 17.5±2.78 mV, n = 9, for ketamine-xylazine and pentobarbital anaesthesia, respectively; p = 0.9089) even though individual mean voltage threshold values could largely differ between individual neurons (Fig. 1E,F). The overall action potential voltage thresholds also remained similar under both types of anesthesia (−46.31±5.23 mV, n = 13, and −47.26±2.88 mV, n = 9, for ketamine-xylazine and pentobarbital, respectively; p = 0.7066). These results show that the voltage threshold for action potentials in TRN neurons is very dynamic, and the wide operational range is robust as it is similarly expressed in two different oscillatory network dynamics.

**Figure 2:**
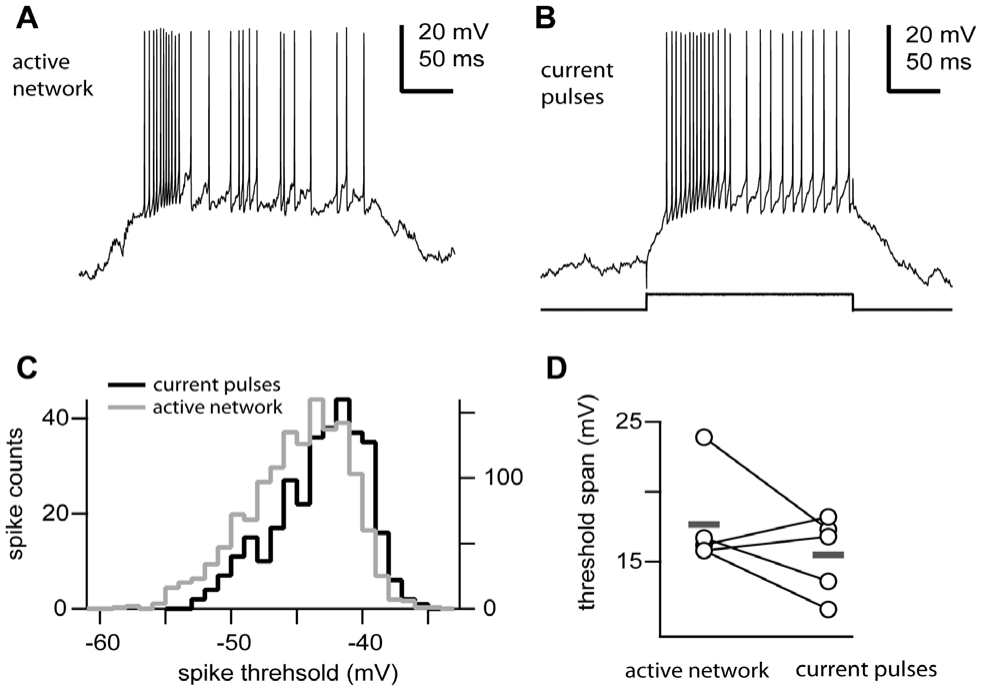
The action potential threshold range is an intrinsic membrane property. In vivo firing patterns of a TRN neuron (ketamine-xylazine anaesthesia) intracellularly recorded during spontaneous synaptic active activity (**A,** active network) or intracellular current injection (**B,** current pulse, +1 nA). **C,** Distribution of voltage thresholds for the previous neuron during both conditions (active network, n = 1404 spikes; current pulses, n = 330 spikes). Note different scales for both conditions. **D,** Action potential threshold range for TRN neurons driven by intracellular current pulses or spontaneous active network episodes. Means were not significantly different (n = 5, p = 0.248). Circles and grey bars show individual values and means, respectively. Scale bars: A, B, vertical 20 mV, horizontal 50 ms.

The fact that the wide range of the spike thresholds found in TRN neurons was similar under two distinct network conditions suggested that it could be the result of intrinsic membrane mechanisms. To establish if that was the case, we compared in the same cells the distribution of action potential thresholds in response to depolarizing currents steps at various intensities (0.5–1.5 nA) to the onset range measured from the spontaneously active periods during network oscillations; that is, activated epochs during slow oscillations (in recordings with ketamine-xylazine anesthesia) or spindle oscillations (in recordings with barbiturate anesthesia) (Fig. 2). The variation (standard deviation) in action potential threshold in response to depolarizing current pulses (3.3±0.6 mV) was statistically not different to that found during spontaneously activated network states (3.0±0.5 mV, n = 5; p = 0.9365); suggesting that the wide operational range of the spike threshold is an intrinsic membrane property not modulated by the synaptic background and ongoing network activity (Fig. 2A,B). The voltage threshold exhibited similar range for both conditions as mean values were not statistically different (15.5±2.79 mV and 17.68±3.5 mV, for current pulses and active network, respectively; p = 0.248, paired two-tailed t test; Fig. 2D). Because of our small number of samples (n = 5) and the large variability seen in the in vivo condition, we performed a bootstrap analysis for both distributions and confirmed that the voltage threshold spans could not be distinguished (bootstrap means 15.52 and 17.66, confidence intervals [12.92 17.46] and [16.04 22.46]; p>0.1, 1000 iterations; for current pulses and activated network conditions; respectively). Furthermore, the distribution of action potential thresholds (mean −46.32±3.82 mV) in response to depolarizing current steps was very similar to that obtained during active periods of spontaneous network activity (mean −47.18±3.23 mV, n = 5; p = 0.5476. Fig. 2C).

Several causes underlie the variation in action potential threshold. Previous studies have found in cortical neurons that the rate of change of the membrane potential preceding the action potential affects the action potential initiation [3], [4], [25], [26], [27]. Neurons have refractory periods that limit their firing rates, and thus the action potential voltage threshold also varies as a function of the instantaneous firing rate. To exclude the influence of short interspike intervals, only spikes separated by at least 20 ms from any preceding spike were considered for this part of the analysis [3], [4] (see Methods). TRN neurons discharge high-frequency bursts (200–400 Hz, [28]), and thus a significant fraction of the discharged spikes was discarded from this analysis. Indeed, the fraction of tonic spikes (separated by at least 20 ms) discharged by TRN neurons accounted for as much as half of the total spike count (53±24%; n = 32576 action potentials, 23 cells).

**Figure 3:**
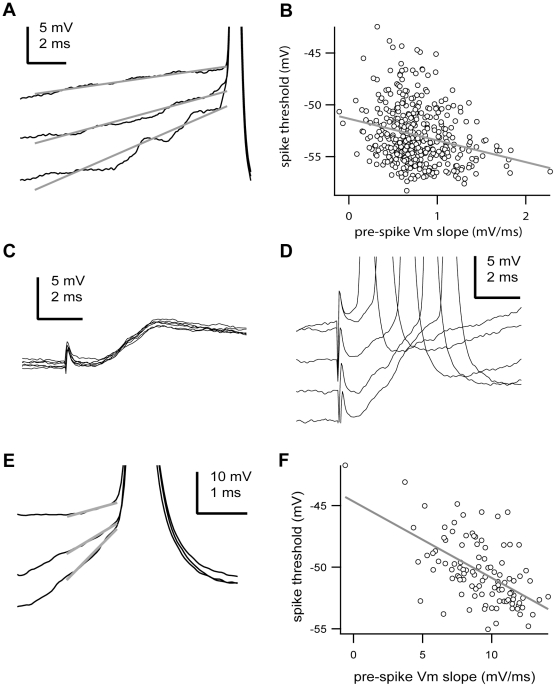
The voltage threshold of action potentials is negatively correlated with the preceding rate of membrane potential depolarization. **A,** Three action potentials (clipped) recorded from a TRN neuron are shown with the membrane potential change leading to the action potential onset. Each recording is shown with its respective best-fit line prior (10 ms) to the action potential onset. Spikes were arbitrarily clipped for illustration purposes only. **B,** Plot of the action potential voltage threshold versus the membrane potential change over the 10 ms preceding each action potential (pre-spike Vm slope) recorded in the cell shown in A. The line is the best-fit line by the equation y(V) = −51.34 mV – 2.09 ms*x(dV/dt), n = 405 spikes, R = −0.2497, p<0.0001, two-tailed t test. **C,** Subthreshold EPSPs triggered by electrical stimulation of a topographically connected cortical area. Note the long-lasting decay of the EPSPs. **D,** Suprathreshold EPSPs followed by action potentials with different latencies in the same cell shown in C. Spikes were arbitrarily clipped for illustration purposes only. **E,** Three action potentials (clipped) triggered by evoked EPSPs are shown with the membrane potential change leading to the action potential onset. Each recording is shown with its respective best-fit line prior (1 ms) to the action potential onset from the cell shown in C. Spikes were arbitrarily clipped for illustration purposes only. **F,** Plot of the action potential voltage threshold versus the membrane potential change over 1 ms preceding each action potential (pre-spike Vm slope) recorded in the cell shown in C. The line is the best-fit line by the equation y(V) = −44.68 mV – 0.62 ms*x(dV/dt), n = 77 spikes, R = −0.5728, p<0.0001, two-tailed t test. Scale bars: A, C,D, vertical 5 mV, horizontal 2 ms; E, vertical 10 mV, horizontal 1 ms. Some portions of raw data (intracellular voltage recordings) from the cells in this figure have been used in previous publications (see Methods).

In the population of analysed tonic spikes, we found results similar to previous studies in cortical cells [3], [4]. That is, the action potential threshold was negatively correlated to the rate of membrane potential change preceding (10 ms) the action potential (Fig. 3A,B). Overall, there was a weak negative (R = −0.26±0.16), yet statistically significant relationship (p<0.05, two-tailed t test) for the majority of cells (82.6%, n = 19 of 23) between the rate of membrane potential depolarization and spike voltage threshold, with faster changes leading to lower thresholds. To test if the detected negative correlation was in fact due to synchronous synaptic inputs we evoked EPSPs in TRN neurons by electrically stimulating corticothalamic fibers in the internal capsula (Fig. 3C,D). We found a negative (R = −0.38±0.18) correlation in all cases (n = 8 afferents pathways in 4 cells) between the rate of membrane potential depolarization produced by the EPSPs and the spike voltage threshold (Fig. 3E,F). The relationship was statistically significant (p<0.01, two-tailed t test) for the majority of cases (75%, n = 6 afferents pathways; the remaining 2 were not significant likely due to the small sample of data points, n = 20–30), with faster membrane depolarizations leading to lower spike thresholds (Fig. 3E,F). Note that spike voltage threshold is related to the preceding membrane potential, and not only its rate of change. The fact that the voltage threshold of action potentials is sensitive to the preceding rate of membrane potential depolarization suggests that TRN neurons might act as coincidence detectors of synchronized synaptic inputs.

**Figure 4:**
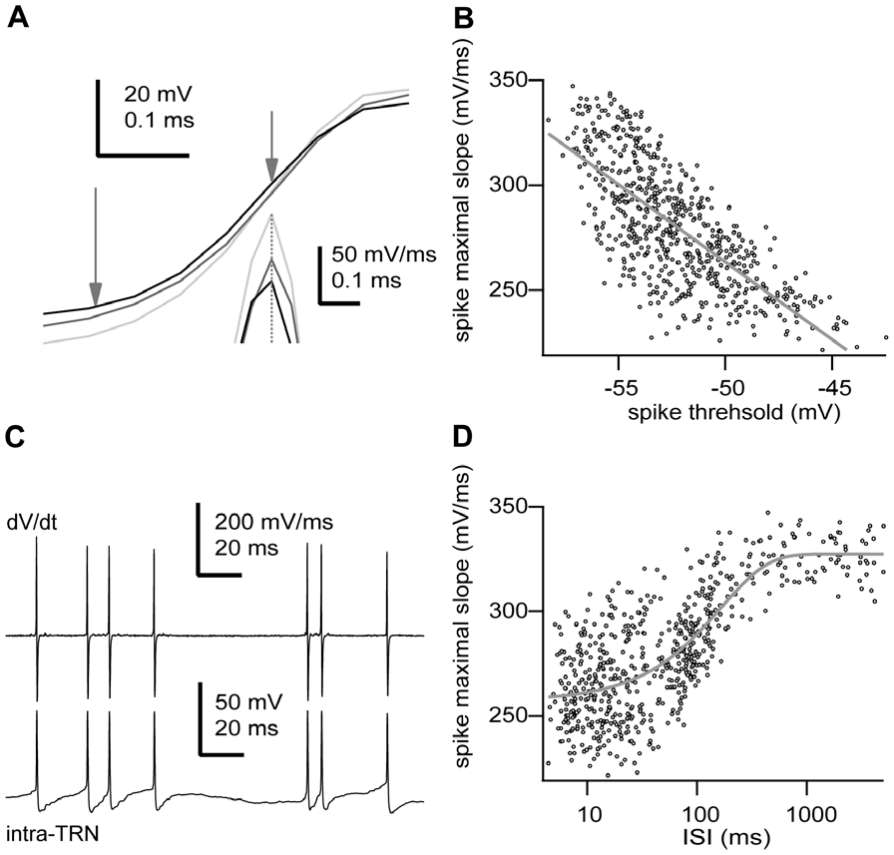
Action potential voltage threshold is negatively correlated with the maximal rate of depolarization during the action potential. **A,** Three different action potentials during the rising phase. Vertical arrows depict the position of the threshold (left) and maximal slope (right). Inset, derivative of the action potential at the point indicated by the arrow. Note different time scale for the inset. **B,** Plot of the action potential maximal rising slope versus voltage threshold for all recorded action potentials (n = 633 spikes). The line is the best-fit line by the equation y(dV/dt) = −105.72 mV/ms – 7.38 ms^−1^*x(V), R = −0.725, p<0.0001, two-tailed t test. **C,** bottom, sequence of action potentials discharged by the cell shown in A. Top, derivative (dV/dt) of the membrane potential (intra-TRN) for the trace at the bottom. Note variation in peak amplitude in the derivative. **D,** Plot of the action potential maximal rising slope versus the inter-spike interval for all recorded action potentials (n = 633 spikes). The line is the best-fit line by the exponential function y(dV/dt) = 327.43 mV/ms+68.5 mV/ms*exp (x(V)/164.66 ms), p<0.05, Kolmogorov-Smirnov test. Scale bars: A, spike, vertical 20 mV, horizontal 0.1 ms; derivative, vertical 50 mV/ms, horizontal 0.1 ms; C, dV/dt, vertical 200 mV/ms, horizontal 20 ms; intra-TRN, vertical 50 mV, horizontal 20 ms. Some portions of raw data (intracellular voltage recordings) from the cell in this figure have been used in previous publications (see Methods).

The increase in the spike threshold could be related to a decrease in the availability of sodium channels due to inactivation [3]. The maximal rate of the action potential rising phase is directly proportional to the maximal sodium current during a spike [25], [29]; and so, a decrease in the maximal rate of rise would indicate an increase in sodium channel inactivation [29]. To test this idea, we calculated the correlation between the action potential threshold and the maximal rising slope of the action potential (fig. 4A,B). Indeed, the action potential threshold was negatively correlated with the maximal rising slope (R = −0.74±0.15 ms, n = 23; p<0.0001, two-tailed t test), consistent with the hypothesis that spike threshold increases as a function of sodium channel inactivation [5], [6], [7], [8]. Again, considering the maximal rate of the action potential rising phase as directly proportional to the maximal sodium current during a spike, we could estimate the time-dependent recovery of inactivation in sodium channels during naturally-occurring network activity patterns (fig. 4C,D). We estimated inactivation from the ratio of decrease in the maximal rate of the action potential rising phase (mean maximal decrease 37.35±9.78%, n = 23, see Methods). For the majority of cells (78.3%, n = 18 of 23), recovery from inactivation followed single exponential kinetics (time constant 180.49±111.67 ms, n = 18, p<0.05, Kolmogorov-Smirnov test. The remaining 5 cells showed a similar trend, yet the fitting was not statistically significant). These results suggest that the voltage- and time-dependent properties of voltage-gated sodium channels shape the sensitivity of TRN neurons to transient episodes of membrane depolarization and the performance of coincidence detection.

**Figure 5:**
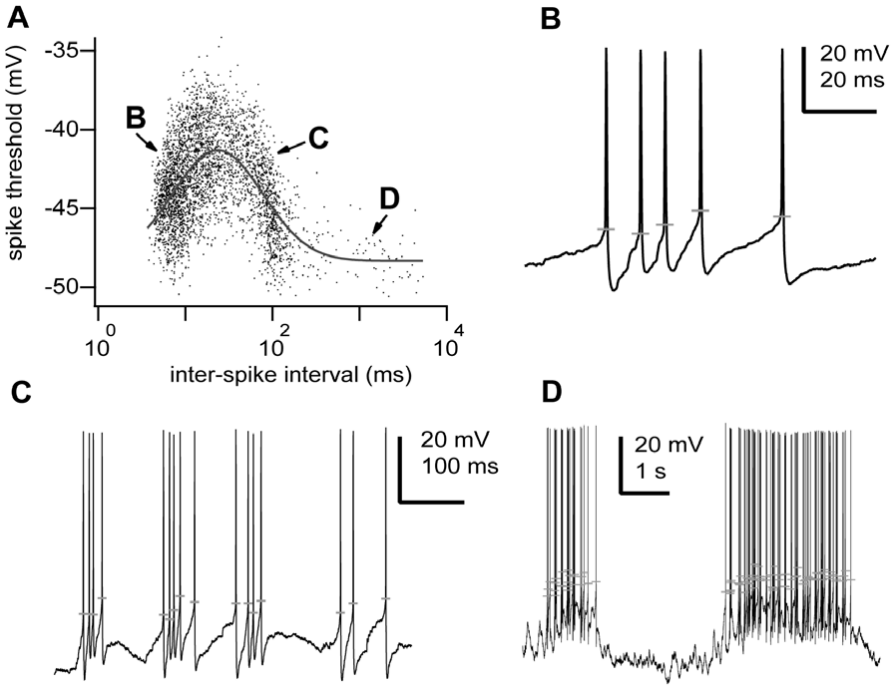
The voltage threshold of action potentials is a function of the preceding spike time interval. **A,** Plot of the action potential voltage threshold versus the time (in logarithmic scale) since the last action potential recorded in a TRN neuron. The line is the best-fit line by the log-normal function y(V) = 48.33 mV+7.03 mV*exp[−(ln(x(t)/23.52 ms)/1.64)^2^], n = 3644 spikes, p<0.001, Kolmogorov-Smirnov test. **B–D,** Three interval ranges in the log-normal distribution correspond to discrete physiological intracellular states of activity. B, high-frequency burst discharge; C, inter-burst discharge periods; and D, inter-network active periods. Grey horizontal lines show for each spike the voltage threshold. Scale bars: B, vertical 20 mV, horizontal 20 ms; C, vertical 20 mV, horizontal 100 ms; D, vertical 20 mV, horizontal 1 s. Some portions of raw data (intracellular voltage recordings) from the cell in this figure have been used in previous publications (see Methods).

Previous modelling studies have suggested that for short intervals, the inter-spike interval (ISI) could influence the occurrence of subsequent spikes [30] due to the potassium channel-mediated refractory period. We also found for short intervals significant effects due to sodium channels inactivation (Fig. 4). In addition, recordings from hippocampal pyramidal neurons in vivo have shown that even long ISIs, as longs as 1 s, can affect the action potential threshold; likely due to the effect of sodium current inactivation [4], [31]. Given these precedents, we looked for possible effects of previous ISI on the action potential threshold. We found for most cells (86.2%, n = 19 of 23) that the action potential threshold was correlated with the time since the last action potential (Fig. 5A; p<0.05, Kolmogorov-Smirnov test). The log-normal relationship was continuous; however, it could be associated to three different stages, which corresponded to particular states of neuronal activity. Thus, for short intervals (ISI<15 ms) or close to the range of high-frequency bursts, each action potential discharged increased the voltage threshold of the following spike making it more depolarized (Fig. 5B). For intermediate intervals (ISI = 15–300 ms) or between high-frequency bursts of the same network active period (i.e., spindles or slow oscillations), action potential thresholds were negatively correlated with the preceding interval (Fig. 5C). Finally, for long intervals (ISI>300 ms) or between network active periods, spike voltage thresholds were weakly or not correlated with the preceding interval (Fig. 5D).

**Figure 6:**
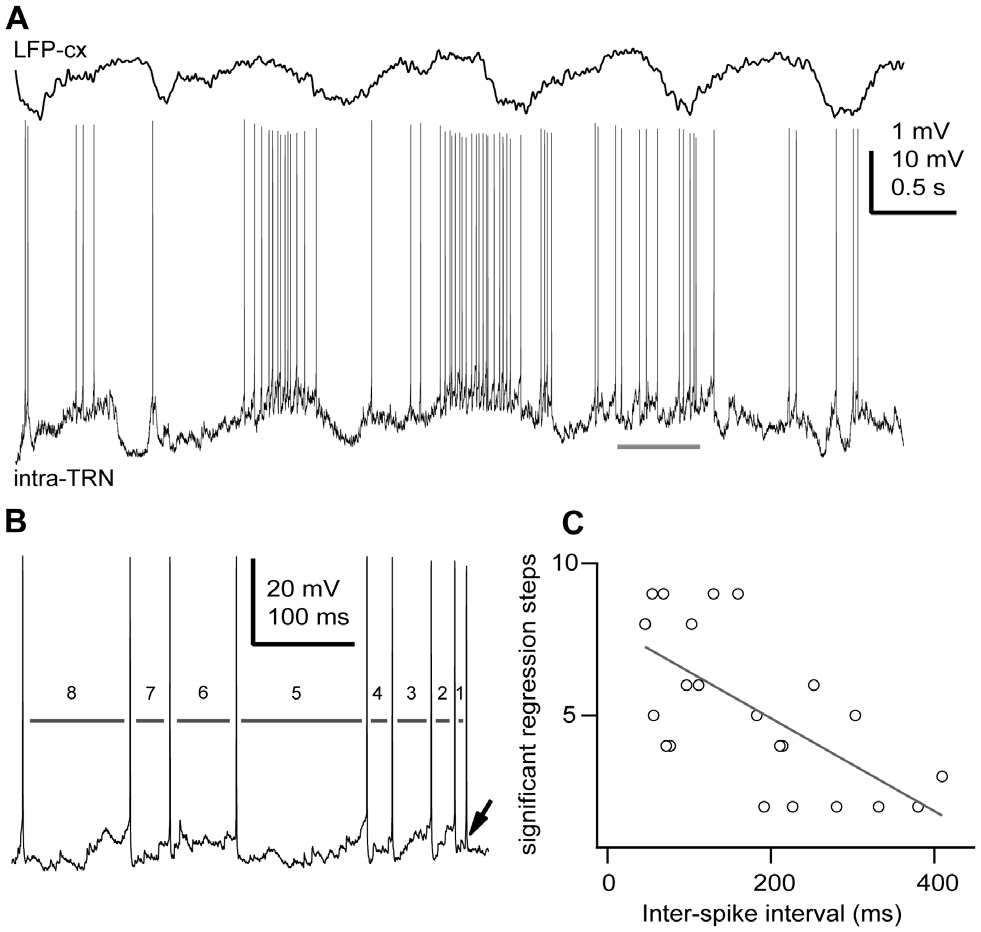
The action potential threshold depends on the occurrence of multiple previous action potentials. **A,** In vivo intracellular recording of a TRN neuron during slow oscillations in the cortex (ketamine-xylazine anaesthesia) shows the large range of inter-spike intervals. **B,** A segment from A (horizontal bar) is expanded for clarity. For this cell, it was found that the threshold of any particular action potential (arrow) is influenced by eight previous ISIs (grey bars). **C,** Summary results for all TRN neurons recorded for this study. Plot of the number of significant partial regression steps (p<0.05) versus the mean ISI for each neuron. Line is the best-fit line by the equation y = −7.96–0.02*x(t), r = −0.8481, n = 23 cells, p<0.0001, two-tailed t test. Scale bars: A, LFP-cx 1 mV, intra-TRN 10 mV, 0.5 s; B, vertical 20 mV, horizontal 100 ms.

To test if more than a single previous action potential could be modulating the initiation of any given action potential, we applied a partial regression analysis on multiple previous ISIs (Fig. 6). The analysis explains the variability in the threshold of a single action potential in relation to the 1st, 2nd, …, *n*th previous ISI. In fact, the action potential threshold of any given spike was partially correlated with multiple previous ISIs. In addition, the number of significantly correlated previous ISIs was linearly correlated with the mean ISI for any given cell (Fig. 6C; R = −0.8481, n = 23 cells, p<0.0001, two-tailed t test). Thus, the higher the firing rate of a neuron, the higher the number of preceding spikes influencing the voltage threshold of any given action potential (Fig. 6C). As there was no significant correlation between the average membrane potential and the mean ISI across the recorded cells (p = 0.2562; n = 23; data not shown), the time interval when action potentials can affect the threshold of any given action potential could be calculated from the product of the number of significant regression steps (range 2–9) and the mean ISI (range 46–410 ms) for each cell [4]. Thus, the mean amount of time when preceding action potentials can influence any given action potential was 742.89±394.01 ms (n = 23). Considering that active periods during slow-wave and spindle oscillations rarely reach 1 s in duration [23], [24], these results could imply that that the first action potential discharged during an active network period might influence the voltage threshold of the last action potential of the same episode.

**Figure 7:**
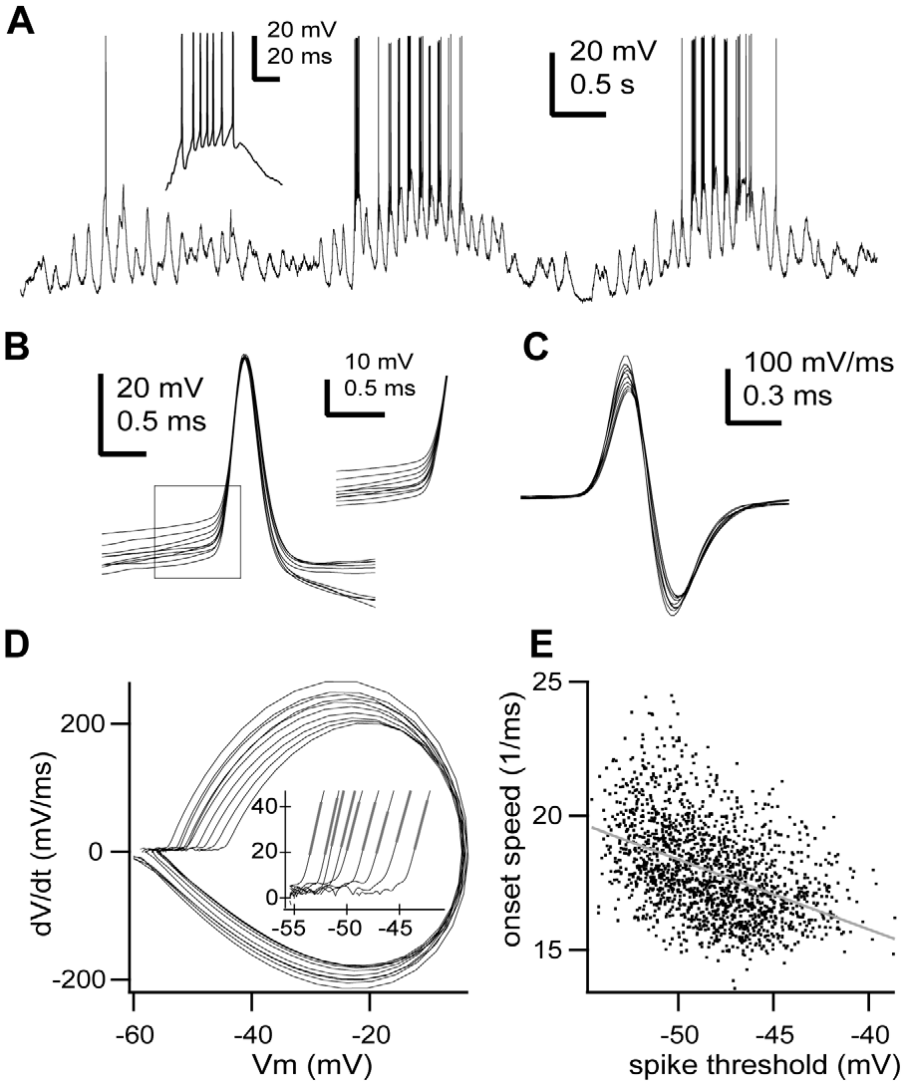
The onset speed of action potentials is voltage-dependent. **A,** In vivo intracellular recording of a TRN neuron during spindle oscillations (pentobarbital anaesthesia). Inset, typical high-frequency burst discharge. **B,** Sample of consecutive action potentials (n = 10) from the cell shown in A. Inset, higher magnification of the action potential onset shows the absence of kink **C,** Derivative of the action potentials shown in B. Note different time scale than B. **D,** Phase plane representation of the action potentials shown in B. Note monophasic rate of rise. Inset, higher magnification of the action potentials onset shows the rapid onset. Grey lines show the linear fits used to calculate the onset speed of the action potentials. **E,** Plot of the action potential onset speed (slope of the initial rate of rise after the threshold (20 mV/ms) in the phase plane) versus voltage threshold. Line is the best-fit line by the equation y(onset speed) = 5.34 mV^−1^ms^−1^ – 0.26*x(Vm), n = 1722 spikes, r = −0.438, p<0.0001. Scale bars: A, vertical 20 mV, horizontal 0.5 s; inset, vertical 20 mV, horizontal 20 ms; B, vertical 20 mV, horizontal 0.5 ms; inset, vertical 10 mV, horizontal 0.5 ms; C, vertical 100 mV/ms, horizontal 0.3 ms. Some portions of raw data (intracellular voltage recordings) from the cell in this figure have been used in previous publications (see Methods).

So far we have characterised the onset range of action potential voltage thresholds. Next, we set out to analyse the onset speed of action potentials. The dynamics of action potential initiation in TRN neurons was characterized by a very rapid onset and a steep slope in membrane potential (Fig. 7), similar to what has been described in detail for pyramidal cells [32], [33], [34]. The rapid onset was apparent in the phase plane plot that graphs the rate of change of membrane potential (dV/dt) against the instantaneous membrane potential (Vm), where it was manifested as a steep vertical take-off at the onset of the trajectory of the action potential (Fig. 7D). In pyramidal cells and other neuron classes, the action potential backpropagates from the axon initial segment (the initiation site) to the soma resulting in the high rate of rise of membrane potential observed at the foot of the action potential [33], [35]. This is seen as a kink in the membrane potential in the time domain and as a biphasic rate of rise in the trajectory of the spike waveform in the phase plane in pyramidal cells and other neuron types. Nevertheless, all recorded TRN neurons exhibited a monophasic rate during the rising phase of somatically recorded action potentials (Fig. 7D), yet the onset speed remained high (18.02±2.94 ms^−1^, n = 23).

**Figure 8:**
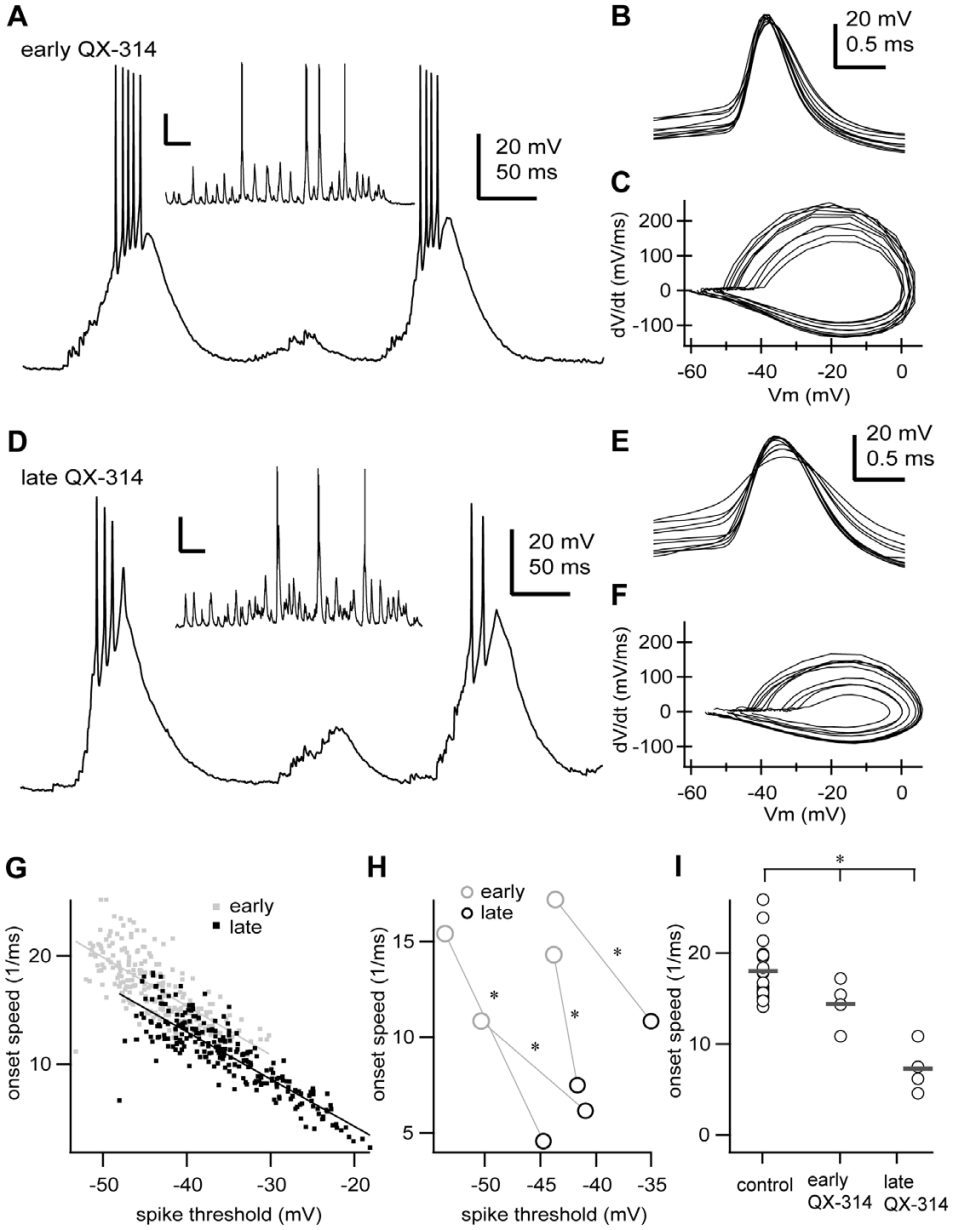
The onset speed of action potentials depends on the effective density of available sodium channels. **A,** In vivo intracellular low-threshold burst discharges of a TRN neuron recorded during the first 10 minutes after impalement with a recording pipette loaded with QX-314 (0.5 M). Inset, typical episode of spindle oscillations from the same cell (pentobarbital anaesthesia). **B,** Sample of consecutive action potentials (n = 10) from the cell shown in A. **C,** Phase plane representation of the action potentials shown in B. **D,** Same cell as in A, recorded 25 minutes after impalement. Inset, example episode of spindle oscillations. **E,** Sample of consecutive action potentials (n = 10) recorded 25 minutes after impalement. **F,** Phase plane representation of the action potentials shown in E. **G,** Plot of the onset speed of action potentials versus the voltage threshold for the cell shown in A–F during the first 10 minutes of recording (early QX-314, n = 212 spikes) and after 25 minutes of recording (late QX-314, n = 254 spikes). **H,** Plot of the mean onset speed of action potentials versus the mean voltage threshold for each of the cells recorded with QX-314. Changes were statistically significant in all cells (p<0.05, two-tailed paired two-tailed t test). **I,** Onset speed of action potentials for all cells recorded in this study. Circles and grey bars show individual values and means, respectively. Control, data from neurons recorded without QX-314; early QX-314, data from neurons during the first 10 minutes of recording with QX-314; late QX-314, data from neurons after 25–50 minutes of recording with QX-314. Control, n = 23 cells, 32576 spikes; early QX-314, n = 4 cells, 1728 spikes; late QX-314, n = 4 cells, 386 spikes. Mean values were significantly different (*, p = 0.0004, Kruskal-Wallis test). Scale bars: A,D, vertical 20 mV, 50 ms; inset, vertical 20 mV, 0.5 s; B, E, vertical 20 mV, 0.5 ms. Some portions of raw data (intracellular voltage recordings) from the cell in this figure have been used in previous publications (see Methods).

Previous modelling studies have predicted that the steepness of the action potential onset in cortical neurons is independent of the spike threshold [32], [34]. Conversely, the onset speed of the action potential was voltage-dependent for most TRN neurons (95.6%, n = 22 of 23), showing a negative correlation with the voltage threshold; so that the more depolarized the spike threshold, the slower the onset speed (Fig. 7E). The voltage-dependency (regression slope = −0.29±0.2 mV^−1^ms^−1^) was statistically significant (R = −0.35±0.2, n = 22, p<0.05, two-tailed t test). Furthermore, reducing the effective density of available sodium channels by QX-314 application in the recording pipette (fig. 8) decreased not only the action potential amplitude (control 50.68±8.34 mV, early QX-314 41.47±3.15 mV, late QX-314 25.97±8.49 mV; p = 0.0007, Kruskal-Wallis test; Fig. 8B,E) and frequency (control 8.98±6.34 Hz, early QX-314 2.98±3.01 Hz, late QX-314 0.68±0.34 Hz; p = 0.0011, Kruskal-Wallis test; Fig. 8A,D), as expected; but also the onset speed in TRN neurons (control 18.02±2.94 ms^−1^, early QX-314 14.4±2.61 ms^−1^, late QX-314 7.27±2.67 ms^−1^; p = 0.0004, Kruskal-Wallis test; Fig. 8C,F,G,H). As time elapsed, the effect of QX-314 deepened and the action potential onset speed became slower (early QX-314 14.4±2.61 ms^−1^, late QX-314 7.27±2.67 ms^−1^; n = 4, p = 0.0118 paired two-tailed t test; Fig. 8I). Nevertheless, the voltage-dependency of the onset speed was not affected by decreasing the density of available sodium channels (control −0.29±0.20 mV^−1^ms^−1^, early QX-314 −0.47±0.25 mV^−1^ms^−1^, late QX-314 −0.51±0.06 mV^−1^ms^−1^; exact p = 0.0559, Kruskal-Wallis test).

These results indicate that the onset speed of action potentials is voltage-dependent in TRN neurons, a phenomenon that also depends on the effective density of sodium channels, as it becomes more apparent when sodium channels are blocked by the lidocaine derivative QX-314.

## Discussion

We have studied the dynamics of initiation of action potentials in the GABAergic TRN neurons in vivo. We found that TRN neurons are sensitive to synchronous synaptic inputs that result in rapid changes in the membrane potential preceding the onset of action potentials, suggesting a dynamic mechanism for synaptic coincidence detection. In addition, we have previously shown that TRN neurons can perform as good rate-coding integrators [19], so TRN neurons might combine the seemingly incompatible functions of coincidence detection and temporal integration.

Our results demonstrate that TRN neurons do not perform only as enhanced temporal integrators that discharge an action potential once the voltage threshold is reached. Instead, spike threshold varies over a wide operational range of membrane potentials and is sensitive to the rate of membrane potential depolarization. So that the spike threshold is lowest when the membrane potential depolarizes rapidly and highest when TRN neurons are depolarized and discharging at high rates. This indicates that the mechanism of spike generation dynamically regulates the neuronal output as a function of the magnitude and time course of the membrane potential depolarization [36]. As expected, volleys of synchronous synaptic inputs produce rapid membrane potential depolarizations as we could demonstrate by triggering EPSPs from electrical activation of corticothalamic fibers. Furthermore, the spike voltage threshold of both spontaneous and evoked action potentials showed similarly negative correlations with the rate of membrane potential depolarization. Thus, the ionic mechanism responsible for action potential generation enhances the sensitivity of TRN neurons to coincident synaptic inputs.

Despite the fact that the TRN nucleus is entirely constituted by GABAergic cells, it is still able to generate and propagate patterned synaptic activity, both locally and globally [37]. Indeed, the TRN can internally trigger precisely-timed spiking in its own neurons and in thalamic relay neurons [11]. Therefore, the TRN can be considered as an encoding element in the sequence of information processing along the sensory pathway. We have previously described how a secondary depolarizing component can produce a long voltage-dependent time constant in cortical EPSPs, which suggests that TRN neurons act as good rate-coding integrators [19]. Thus, TRN neurons in vivo potentially combine the functions of coincidence detection and temporal integration, which have been regarded as incompatible [20], [21]. Interestingly computational models based on detailed reconstructed cat neocortical pyramidal cells have shown that the synaptic background could act as a switch between integrations modes, favouring temporal integration or coincident detection depending on the synaptic context [38]. In addition, the switching between temporal integrator or coincidence detector modes could depend upon the fraction of sodium channels available in the axon initial segment after different activated states, considering that its density could reach several orders of magnitude over the soma [39]. For example, variations in the density of sodium channels in the axon initial segment in the magnocellularis nucleus of birds have been reported to adjust and optimize for coincident detection of different auditory frequencies [40].

Our results show that TRN neurons exhibit one of the most dynamic spike voltage thresholds reported so far; a fact suggesting that these neurons can flexibly integrate synaptic inputs to discharge action potentials over wide voltage ranges. The GABAergic nature of TRN neurons does not explain the large spike voltage thresholds variability reported here, as previous studies have found narrow dynamic ranges for GABAergic neurons in the neocortex [25], [32] and hippocampus [41]. The high level of expression of the powerful calcium-mediated low threshold spike can underlie the large dynamic range of voltage thresholds as it is similar to the large variability reported for intrinsically bursting cells in the neocortex (16.4±1.4 mV, reported in [25]; however, lower values, 10 mV, were reported in [32]), but largely superior to other bursting cells, such as pyramidal neurons in the hippocampus (5.7±1.7 mV, [4]). This idea is further supported by the observation that the large variability detected in the voltage threshold was an intrinsic membrane property, and not the result of a particular oscillatory network state or synaptic pattern of activity. Indeed, we detected similar voltage threshold ranges during distinctively different network rhythms –spindle oscillations and slow waves, which are characteristic of particular stages of sleep [42]. Further experiments will have to assess if similarly wide operational ranges of voltage thresholds are present in activated states, such a waking or rapid eye movement (REM) sleep, when spontaneous extracellular voltage fluctuations are much smaller and synaptic inputs are mostly asynchronous [43], [44].

The large variability of voltage thresholds is unlikely to be relevant for the precise timing of burst discharges, as they activate large GABA_B_-receptor mediated IPSPs onto target thalamocortical cells that overcome GABA_A_-receptor mediated inhibitory potentials [45]. Such large IPSPs can be considered as single events and are most likely unaffected by minor changes in the discharge frequency (>100 Hz) of the presynaptic TRN terminal. However, TRN neurons also discharge tonic spikes at lower frequencies that activate exclusively GABA_A_-receptor mediated inhibitory currents [46]. Under such regime, the spike timing might be critically important as it has been shown in other areas, such as the neocortex or the hippocampus [47], [48]. Furthermore, the preservation of synaptic delays between inhibition and excitation and accurate spike timing of distinct neuronal classes is critical for the maintenance of optimal operations and functioning of the neural circuits, for even millisecond-changes can distort the fine network of temporally structured activity, producing detrimental effects.

During wakefulness, when most sensory information is encoded and relayed to the cortex, TRN neurons operate mostly, if not exclusively, under the tonic regime. Under such conditions, the dynamic range of spike thresholds could be highly relevant for setting the precise timing of inhibition on thalamocortical cells, gating the flow of information between the thalamus and the cortex. During sleep stages, when large territories are highly synchronized and organized by spindle oscillations and slow waves, thalamocortical neurons and corticothalamic intrinsically bursting neurons preferentially discharge high-frequency bursts (>200 Hz), impinging onto TRN neurons and driving their activity [49]. Such bursts of action potentials could be detected, discriminated and encoded by the coincidence detection mechanism that we describe here. On the other hand, during waking states and REM sleep, thalamocortical and regular spiking corticothalamic neurons discharge in tonic mode at relatively low frequencies (<50 Hz), which detection could be favoured and enhanced by the long membrane time constant that we have previously described [19]. Thus, TRN neurons might be endowed with specific mechanisms to selectively integrate and process synaptic inputs according to the state of vigilance.

Recordings from pyramidal neurons have shown a biphasic rate of rise during early phases of the action potential [33]. The initial phase reflects the spike initiation in the axon initial segment (kink), whereas the subsequent phase represents the somatodendritic propagation [35]. Such trajectory seems to be dependent on the anatomical preservation of the axo-dendritic arbor because the kink is absent in neurons recorded in slices [33]. Nonetheless, we found monophasic rates of rise, and thus no kink, in all recorded TRN neurons, where the axo-dendritic structure is preserved and naturally-occurring spatiotemporal patterns of synaptic activity take place. This fact might be related to the GABAergic nature of TRN neurons, since other GABAergic cells have been reported to exhibit monophasic rates of rise in slices, like hippocampal basket cells [41]. However, other neuronal populations have also been reported to exhibit monophasic rates of rise, such as subthalamic nucleus neurons [9] o even cortical pyramidal neurons [32]. Much work has been concentrated on pyramidal cells regarding the dynamics of action potential initiation. Some of the features reported here might be related to the GABAergic nature of TRN cells, and should be present in other GABAergic cell classes. However, the field has received little attention and further experimental studies will have to address this important issue in the future.

Previous studies have provided experimental and modelling evidence for the steepness of the action potential onset in cortical neurons as independent of the spike threshold [32]. Indeed, changes in the effective peak sodium conductance -mimicking inactivation [4], [25], [32]- leave the steepness of action potential onset unaffected, but shifts the onset potentials in some Hodgkin-Huxley-type models. Conversely, we found that the action potentials discharged by the same TRN neuron exhibited varying onset speeds, which were linearly correlated with the spike threshold. Future experiments, with voltage clamp control and appropriate in vitro environmental conditions will have to be designed to further clarify this important point and identify if this observation extends to other neuronal populations.

Finally, the TRN is not the first structure proposed to combine the seemingly antagonistic functions of temporal integration and coincidence detection. Indeed, recent work in vitro has shown that neurons in slices of the subthalamic nucleus exhibit a balance between active and passive conductances that gives them an effectively zero membrane conductance (and an effectively infinite membrane time constant) over a wide voltage range [9]. Such property argues for subthalamic nucleus neurons operating as temporal integrators. In addition, subthalamic nucleus neurons in vitro exhibit a dynamically changing threshold that enhances sensitivity to synchronous synaptic inputs, supporting the idea of temporal integration [9]. The dynamic spike threshold observed in TRN neurons is likely to be the result of the threshold accommodation phenomenon described in various preparations [5], [27]. In cortical neurons, dynamic spike threshold changes have been linked to stimulus selectivity [50] and to promote coincident detection in the hippocampus [4], subthalamic nucleus [9] and neocortex [3], [25]. We have further shown in vivo, under conditions that preserve anatomically-intact axo-dendritic distributions and naturally-occurring spatiotemporal patterns of synaptic activity, that the GABAergic TRN neurons can operate as both coincidence detectors and temporal integrators, at least in part due to a dynamic spike threshold. This mechanism could be fine-tuned to enhance performance under specific brain states.

## Methods

### Surgery

All experiments were carried out in strict accordance with the recommendations in the Guide for the Care and Use of Laboratory Animals of the National Institutes of Health. Protocols (Sleep rhythms leading to plasticity processes: # 2002-203, # 2003-215, # 2004-219, and # 2004-219-2) were approved by Comité de protection des animaux de l'Université Laval, Vice-rectorat à la recherche et à la création (Quebec, Canada). All surgery was performed under deep anesthesia, and all efforts were made to minimize suffering. Experiments were conducted on adult cats (2.5–4.5 kg) from either gender, anesthetized with pentobarbital sodium (25 mg/kg, i.p.) or a mixture of ketamine HCl or xylazine HCl (10–15 and 2–3 mg/kg, i.m., respectively). When cats showed the signs of deep anesthesia, they were paralysed with gallamine triethiodide (Sigma, St. Louis, MO, USA) and artificially ventilated with control of the end-tidal CO_2_ concentration at 3.5%. Body temperature was maintained at 36–38°C. The depth of anesthesia was continuously monitored by EEG, and additional doses of anaesthetic were administered at the slightest tendency toward low-voltage and fast EEG rhythms. At the end of experiments, animals were given a lethal dose of pentobarbital (50 mg/kg).

### Intracellular recordings and electrical stimulation

Current-clamp intracellular recordings from the rostral and rostrolateral sector of the TRN were performed using sharp electrodes, glass micropipettes (DC resistance, 30–60 MΩ, World Precision Instruments, Sarasota, FL, USA). To avoid breaking of recording micropipettes, the cortex and white matter overlying the head of the caudate nucleus were removed by suction. Pipettes were then descended 3 mm through the caudate nucleus to reach the TRN nucleus. Pipettes were generally filled with a solution of K-acetate (3 M) and, in some experiments, the lidocaine derivative N-ethyl lidocaine (QX-314, 50 mM; Sigma) was added. The stability of intracellular recordings was ensured by cisternal drainage, bilateral pneumothorax, hip suspension, and by filling the hole over the thalamus with 4% agar solution. A high-impedance amplifier with active bridge circuitry (Neurodata, West Warwick, RI, USA) was used to record and inject current inside the cells. Input resistance and intrinsic firing patterns were assessed by using square wave current pulses (range −2 to +2 nA). Electrophysiological signals were digitized at a rate of 20 KHz and stored for off-line analysis.

Electrical stimulation of corticothalamic fibers was performed by descending one or two bipolar stimulating electrodes (Frederick Haer, Bowdoinham, WA, USA) to the internal capsule (anterior +13 mm, lateral +3.5 mm, depth +1 mm) and applying extracellular current pulses (0.2 ms, 50–600 µA, 0.5–1 Hz).

### Data Analysis

The software package Neuromatic (Jason Rothman, University College of London) was used for most analyses. Neuromatic runs in Igor Pro 6.0 (Wavemetrics, Lake Oswego, OR, USA). For some analyses we also used MatLab (MathWorks, Natick, MA, USA). The aim of the analysis was to measure the voltage threshold of each action potential in each cell and to determine the threshold range and onset speed of action potentials and their relation to several parameters of cellular activity. Only cells with large action potentials (that reached 0 mV) that did not decrease in amplitude (>10 mV) during sustained depolarization were included [25]. A total of 27 cells were considered for analysis (ketamine-xylazine anaesthesia, n = 13; pentobarbital anaesthesia, n = 14).

To measure the action potential threshold we used two different methods. In the first method, voltage threshold was defined as the voltage at the onset of each action potential that generated the maximum curvature with a fitting Boltzmann function (−47.19±4.77 mV, n = 27, [51]). In the second method, we defined the spike threshold as the membrane potential at which dV/dt of the action potential exceeded 20 mV/ms in the phase plane (−47.34±5.07 mV, n = 27, [32], [33]). The criterion for spike threshold detection at 20 mV/ms was purely empirical as it was just above the maximal level of dV/dt seen during spontaneous sub-threshold activity. Fixing the threshold at higher or lower levels near this criterion value did not alter the basic results. Both detection methods produced similar results (p = 0.9282), and therefore, they were used indistinctively throughout the study.

In order to detect the influence of the membrane depolarization prior to an action potential on the spike voltage threshold (Fig. 3), we analysed only spikes separated by at least 20 ms from any preceding spike so as to prevent distorting effects from ionic conductances activated by preceding action potentials [3], [4]. Since TRN neurons commonly discharge high-frequency bursts, a potentially large fraction of the discharged spikes would not qualify as tonic spikes. In fact, the fraction of tonic spikes (separated by at least 20 ms) discharged by TRN neurons accounted for about half of the total spike count (53±24%; n = 32576 action potentials, 23 cells). For the rest of the study and all other figures we considered all spikes (tonic and burst) for analysis.

The rate of membrane potential change preceding (10 ms) the action potential was plotted against the voltage threshold of the action potential and correlated by adjusting a linear fit as previously reported for pyramidal cells [4]. Changing the time interval to higher or lower levels near this criterion value did not alter the basic results. In the case of spikes evoked by electrical stimulation the time interval for the membrane potential change preceding the action potential was fixed at 1 ms due to the presence of a large electrical artefact closely preceding de action potential (Fig. 3D).

To obtain an estimation of the degree of inactivation during spiking in vivo (Fig. 4) we calculated for every cell the ratio between the range (arithmetical difference between maximal and minimal values) of the distribution of maximal rising slopes of the action potential and the maximal value of maximal rising slopes of the action potential, and expressed it as percentage. The average value of such quantity across all cells yields the mean maximal decrease.

Some portions of raw data (intracellular voltage recordings) from several of the cells presented here (n = 11 of 27) have been used in previous publications (cell in fig. 7 in PNAS USA 101(26):9816–9821, 2004; cells in figs. 1A and 3C in EJN 20(1):111–119, 2004; cells in figs. 5 and 8 in EJN 20(10):2691–2696, 2004; cells in figs. 1B, 3A and 4 in Thal & Rel Syst 3(1):53–62, 2005). However, the subject of the present study -the dynamics of action potential initiaition- has not been reported in previous studies, thus all data, analysis, results, and figures presented here are original.

### Statistics

Unless stated, all tabulated data are presented as the mean ± SD. Exact Mann Whitney U-test was used for statistical comparisons between populations and the two-tailed t test distribution was used to assess significance of linear correlations. Significant differences were accepted at p<0.05.

In order to compare the means from small samples (fig. 2) we used the resampling bootstrap algorithm [52] The boostrap algorithm accurately estimates an estimator, mean in this case, from a sample by iterative resampling with replacement of the original data. Thus, we computed the mean from the sampled distributions and the difference between distributions, and then resampled with replacement for 1000 iterations. In each iteration the estimator (mean) was computed and the distribution histogram for the resampled estimator was plotted. Since the plotted histogram did not reflect a normal distribution, its mean was determined non-parametrically with a 95% confidence interval (p<0.05 statistical significance).
